# A bacteriocin-based coating strategy to prevent vancomycin-resistant *Enterococcus faecium* biofilm formation on materials of interest for indwelling medical devices

**DOI:** 10.1016/j.bioflm.2024.100211

**Published:** 2024-07-03

**Authors:** Christian Kranjec, Jills Puthiaparambil Mathew, Kirill Ovchinnikov, Idowu Fadayomi, Ying Yang, Morten Kjos, Wen-Wu Li

**Affiliations:** a Laboratory of Microbial Gene Technology, Faculty of Chemistry, Biotechnology and Food Science. Norwegian University of Life Sciences, 1430, Ås, Norway; b School of Pharmacy and Bioengineering, Guy Hilton Research Centre, Keele University, Stoke-on-Trent, ST4 7QB, UK

**Keywords:** Antibiotic resistance, Bacteriocins, Biofilm, Enterococcus faecium, PEEK, Titanium, Stainless steel, Urinary catheter

## Abstract

The ever-increasing use of exogenous materials as indwelling medical devices in modern medicine offers to pathogens new ways to gain access to human body and begin, in some cases, life threatening infections. Biofouling of such materials with bacteria or fungi is a major concern during surgeries, since this is often associated with biofilm formation and difficult to treat, recalcitrant infections. Intense research efforts have therefore developed several strategies to shield the medical devices' surface from colonization by pathogenic microorganisms. Here, we used dopamine as a coupling agent to coat four different materials of medical interest (plastic polyetheretherketone (PEEK), stainless steel, titanium and silicone catheter) with the bacteriocins, enterocin EJ97-short and the thiopeptide micrococcin P1. Water contact angle measurements and x-ray photoelectron spectroscopy were used to verify the effective coating of the materials. The effect of bacteriocins coated on these materials on the biofilm formation by a vancomycin resistant *Enterococcus faecium* (VRE) strain was studied by biofilm-oriented antimicrobial test (BOAT) and electron scanning microscopy. The *in vitro* biocompatibility of bacteriocin-modified biomaterials was tested on cultured human cells. The results demonstrated that the binding of the bacteriocins to the implant surfaces is achieved, and the two bacteriocins in combination could inhibit biofilm formation by *E. faecium* on all four materials. The modified implant showed no cytotoxicity to the human cells tested. Therefore, surface modification with the two bacteriocins may offer a novel and effective way to prevent biofilm formation on a wide range of implant materials.

## 1 Introduction

Advances in material and manufacturing technologies (i.e. 3D printing) made the use of indwelling medical devices a common practice in clinical settings [1,2]. Such devices can be either permanent, such as prosthetic joints, bone replacement prosthetics, cardiac valves, breast implants and so on, or temporary such as urinary catheters and central venous catheters. It is not surprising that the increase in the use of indwelling medical devices also coincided with infections that stem from their bacterial colonization. Consequently, device-associated infections became one of the most compelling contributors to hospital-acquired infections [3]. Such infections, when not treatable, can lead to the surgical removal of the implant with increased sufferance for the patients and costs for health systems [4,5]. Device-associated infections are complicated by the fact that i) they are often associated with biofilm formation onto the indwelling material, ii) there is a high prevalence of antibiotic-resistant bacterial strains in nosocomial environments and iii) biofilms/infections are often polymicrobial. Both Gram-positive and Gram-negative bacteria are responsible for medical device-associated infections. Among the former group, staphylococci (*Staphylococcus aureus* and *Staphylococcus epidermidis*) are responsible for the majority of device-associated infections, whereas enterococci (*Enterococcus faecalis* and *Enterococcus faecium,* in particular) are emerging as important hospital-acquired and medical device-associated pathogens due to the spread of vancomycin- and penicillin-resistant strains [6].

According to the National Institute of Health, biofilms account for the vast majority (≈80 %) of microbial infections in humans (programme calls PA-03–047, PA-06–537). These are formed by sessile bacterial communities in which bacteria produce a protective environment against adverse conditions (i.e. the immune system, antibiotics/antimicrobials). The formation of biofilms is associated with a radical modulation of the metabolic and gene expression patterns as well as deposition of extracellular polymeric substances shielding the bacteria from the external environment. Both staphylococci and enterococci share the ability to form biofilms, which critically contributes to their resilience to antimicrobial treatment. Together with the high prevalence of antibiotic-resistant strains in the two genera, their biofilm-associated infections represent a formidable threat to human health.

In the antibiotic resistance era, research had the urge to develop alternative antimicrobials (e.g., antimicrobial peptides (AMPs)) active on antibiotic-resistant bacteria. Among such AMPs, bacteriocins, peptides produced by bacteria to kill other bacteria, have gained interest due to their amenability and pattern of antimicrobial activity [7,8]. Being ribosomally expressed, many bacteriocins can be easily modified by genetic engineering, they are active against target bacterial species at low concentrations, irrespective of the antibiotic-resistance profile, and they are generally considered not toxic for humans or animals [7,8]. In addition, bacteriocins have also been shown to be useful to eradicate bacterial biofilms and to prevent resistance development when used in combination with other bacteriocins or with antibiotics [[9], [10], [11]]. Many bacteriocins have been shown to be active on medically relevant bacteria. For example, lantibiotic bacteriocins, where nisin is the best studied representative, have a broad spectrum of activity against multidrug-resistant (MDR) species, including methicillin resistant *S. aureus* (MRSA), vancomycin-resistant enterococci (VRE), vancomycin-intermediate *S. aureus* (VISA) and *S. pneumoniae* among others [12]. Furthermore, the combination between the thiopeptide micrococcin P1 (MP1) and the leaderless, non-modified class II bacteriocin garvicin KS was found to synergise with antibiotics against MRSA [9,10,13] whereas the leaderless, non-modified enterocins K1 and EJ97 were effective against *E. faecium* and *E. faecalis*, including nosocomial VRE strains [14,15].

The coating of implantable materials with conventional antibiotics has raised questions about safety, toxicity and fostering of antibiotic resistance among pathogenic bacteria [16]. Other strategies for preventing or removing biofilms from implants have been explored, which include the use of AMPs or physico-chemical treatments, adhesive polymeric coatings, contact killing coatings [16,17]. Implant coating with AMPs demonstrated to be an effective approach to prevent biofilm formation [18,19], although concerns about their toxicity, tissue damage or long-term effects still remain [20,21].

**Fig. 1:**
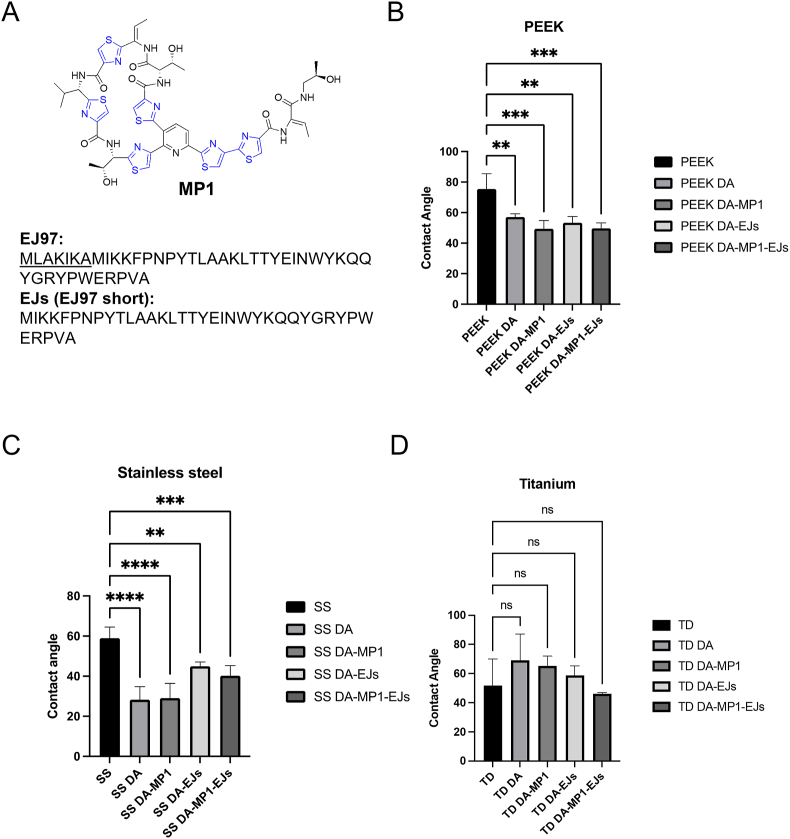
The wettability properties of PEEK, stainless steel (SS) and titanium (TD) discs after coating with dopamine (DA) or DA-bacteriocins (A) Chemical structure of MP1 [35] and sequence of the unmodified bacteriocins EJ97 and EJs [36]. The modified thiazole rings in the MP1 structure are highlighted in blue. B). Water contact angle measurements performed on PEEK discs left uncoated (unmodified PEEK) or coated with polydopamine (DA) or DA and the indicated bacteriocins. Statistical significance was analyzed by pairwise comparison of each group with the unmodified PEEK control. (C and D) Same as in panel 'B' but the water contact angle analysis was performed on stainless steel (SS) (C) or titanium (TD) (D) discs. Shown are the average values (±s.d.) of at least three independent experiments. **p < 0.01; ***p < 0.001; ****p < 0.0001; ns = not significant.

Our previous studies investigated the possibility of using a two-step coating reaction to functionalize stainless steel discs with antibiofilm synthetic peptides [22] and plant-derived cyclotides [23] by exploiting the self-polymerization ability of dopamine [24]. In this study we were interested to test whether bacteriocins could serve as new antibiofilm agents when coated on commonly used materials for medical implants. Titanium and stainless steel as well as polyetheretherketone (PEEK) plastic materials are widely used in orthopedic implants as prosthetic devices [[25], [26], [27], [28]] and/or in dentistry [[29], [30], [31], [32]], whilst silicone (Foley) catheters are the most widely used indwelling urinary catheters worldwide [33,34]. Here, bacteriocins were coated onto the aforementioned materials of medical interest. Using a combination of chemical-physical assays, biofilm viability tests and scanning electron microscopy we show that when in combination, the bacteriocins MP1 and a shortened version of enterocin EJ97 (EJs) (Fig. 1A) can inhibit the biofilm formation by *E. faecium* on the surfaces of four different medically relevant materials.

## 2 Results

### 2.1 Generating bacteriocin-coated materials via the polydopamine coupling strategy

The dopamine self-polymerization methodology was used to functionalize the surface of four different materials (PEEK, stainless steel, titanium and silicone catheter) with the two bacteriocins MP1 and EJs (alone or in combination) (Fig. 1A), where the polydopamine (DA) moiety serves as a bridging agent between the bacteriocins and the surfaces. As shown in Fig. S1, we observed that upon reaction with dopamine, the treated surfaces were covered by a dark-coloured layer. This was particularly evident for PEEK and silicone catheter. To confirm the successful surface modification with dopamine polymerization products, or dopamine-bacteriocin moieties, we assessed the materials' properties by water contact angle measurements and X-ray photoelectron spectroscopy (XPS) analysis. As can be seen in Fig. 1B, the contact angles of PEEK modified with dopamine alone (PEEK-DA) or together with MP1 (PEEK-DA-MP1), EJs (PEEK-DA-EJs), and a combination of MP1 and EJs (PEEK-DA-MP1-EJs) decreased compared to the unmodified PEEK, which indicated that the functionalization with dopamine or dopamine-bacteriocin significantly increased the wettability of PEEK. Decrease of contact angles for these modifications on stainless steel discs were also observed (Fig. 1C). In contrast, the coating procedure yielded non-significant results in the case of titanium discs (Fig. 1D). Such an effect on titanium, however, is in line with results obtained by others [37,38]. Due to the curved structure of the silicone catheter, contact angles of bacteriocin-modified catheters were not measured. Since dopamine is a hydrophilic molecule, this result suggests that, at least for PEEK and stainless steel, the materials' surfaces were successfully modified during the coating procedure. In addition, this might also indicate that the hydrophilic character of dopamine predominates during surface modification, since the strong hydrophobicity of MP1 [39,35] did not significantly impact on the wettability of the dopamine-coated materials.

**Fig. 2:**
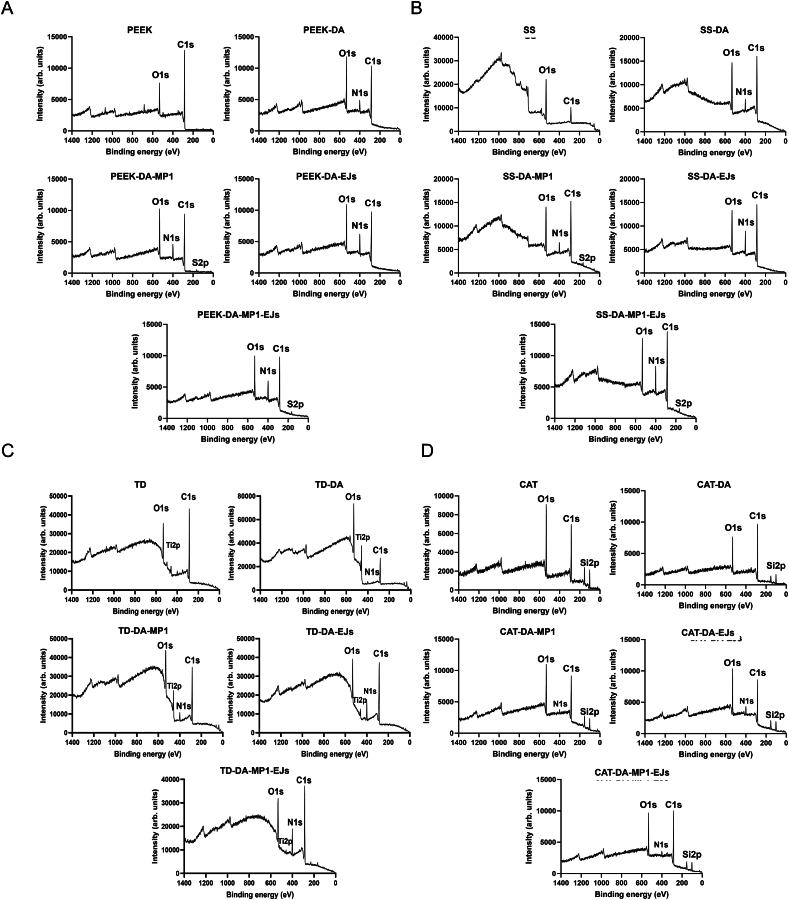
Coating of the indicated materials with dopamine or dopamine-bacteriocins alters their chemical compositions. (A) X-ray photoelectron spectroscopy (XPS) analysis of PEEK discs left uncoated (PEEK) or coated with polydopamine (DA) or with the indicated DA-bacteriocin combinations (MP1, EJs or MP1-EJs). (B–D) Same as in panel A but the XPS analysis was performed on (B) stainless steel discs (SS), (C) titanium discs (TD) or (D) silicone catheter segments (CAT). See also Table S1 for a summary of the presence/absence of peaks N1s and S2p in the different samples.

To obtain further insights on the binding of the bacteriocins to the dopamine-coated materials, we collected XPS spectra of the materials coated either with dopamine alone or in combination with the bacteriocins MP1 and/or EJs. The spectra in Fig. 2A–C shows that coating of with DA introduced the characteristic N1s peaks at 399.8–400.2 eV on PEEK, stainless steel and titanium discs, respectively, which result from the presence of amino groups in the DA and bacteriocins. In contrast, the N1s peak at around 400 eV was very weak upon dopamine coating of silicone (Foley) catheters (Fig. 2D and Table S1). The addition of MP1 or EJs to the DA-coated materials further increased the N1s peaks, indicating a cumulative dose-dependent effect of nitrogen functional groups on the bacteriocin-coated catheters. In addition, the presence of the thiopeptide MP1 introduced a new, weak S2p peak at 160–166 eV on all discs modified with MP1 (Fig. 2, Fig. S2 and Table S1), due to the presence of multiple thiazole rings in its structure [40]. Similar results were also obtained when the two bacteriocins (MP1 and EJs) were used in combination in the coating reaction.

Taken together, these data indicate that dopamine can be successfully employed to mediate the binding of antimicrobial peptides on four types of medically relevant materials of different nature and composition.

### 2.2 MP1 and EJs are effective at inhibiting biofilm formation by E. faecium on different materials of medical interest

Among the nine *E. faecium* strains used in this study, we identified two strains with moderately good biofilm formation abilities: *E. faecium* LMGT 3160 and *E. faecium* LMGT 7660 (Fig. S3), with the latter being a VRE strain (Fig. S4A). These two strains were therefore selected to perform biofilm-related experiments on bacteriocin-coated materials. MP1 and EJs showed antimicrobial activity against the LMGT 7660 and LMGT 3160 when used in a spot-on-lawn assay (Fig. S4A). To explore the ability of bacteriocins to inhibit their biofilm formation on abiotic surfaces we used an adapted version of the biofilm-oriented antimicrobial test (BOAT), in which the metabolic indicator triphenyl tetrazolium chloride (TTC) was used to monitor the metabolic activity retained by biofilm-associated cells after antimicrobial treatment [9,41]. In preliminary assays, LMGT 7660 was used to form biofilms on stainless steel discs coated with an extended set of bacteriocins selected for their broad spectrum of activity against Gram-positive bacteria (MP1 and nisin Z) or for their specificity against enterococcal species (enterocins K1 (K1), EJ97 and EJs) (Fig. S4B). As can be observed, MP1 and EJs showed the best inhibitory activity against biofilm formation by the vancomycin-resistant *E. faecium* strain. Conversely, the bacteriocins nisin Z, K1 alone or in combination with EJ97 failed to significantly impact on the ability of LMGT 7660 to colonize the coated stainless steel discs (Fig. S4C).

**Fig. 3:**
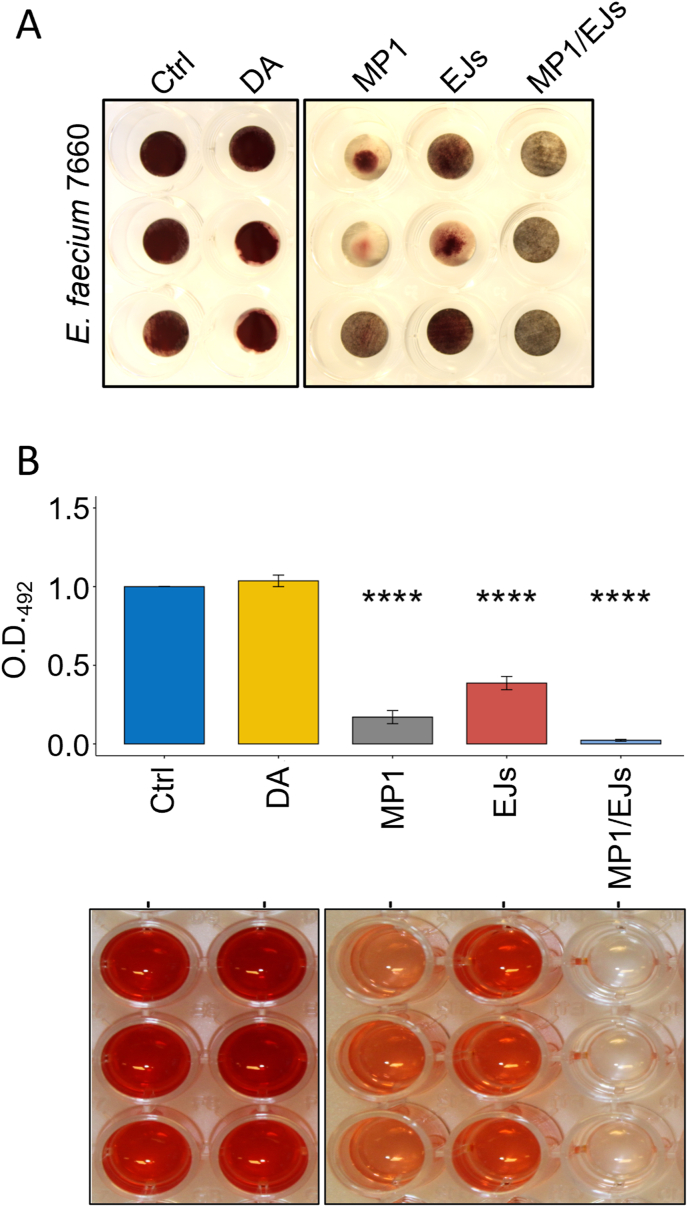
The combination between MP1 and EJs abolishes the biofilm-associated metabolic activity by *E. faecium* LMGT 7660 on stainless steel discs. (A) representative image of stainless steel (SS) discs uncoated (Ctrl), coated with dopamine (DA) or with the indicated bacteriocins after incubation with the metabolic indicator. (B) Representative picture of the red formazan elution product (lower panel) with the respective metabolic activity quantification by optical density readings at 492 nm (O.D._492_ – bar-plot in the upper panel). The metabolic activity is expressed as O.D._492_ fold change relative to the uncoated control (Ctrl). Shown are the average values (±s.d.) of at least three independent experiments. Statistically significant differences in the average metabolic activity across the groups were analyzed with the One-way ANOVA test. Post-hoc pairwise comparisons relative to Ctrl were performed using the two-sample *t*-test. **p < 0.01; ****p < 0.0001. (For interpretation of the references to colour in this figure legend, the reader is referred to the Web version of this article.)

These results prompted us to further investigate the antimicrobial and antibiofilm effects of MP1 and EJs against VRE *E. faecium*. The spot-on-lawn assay suggested a slightly enhanced effect of combining the two bacteriocin (Fig. S4A). In fact, MP1 and EJs were shown to act synergistically in broth cultures against both *E. faecium* LMGT 7660 and LMGT 3160 (fractional inhibition index (FIC) < 0.07, Table S2). We therefore tested whether the two bacteriocins also could have a cumulative inhibitory effect when used in combination to coat stainless steel discs. As shown in Fig. 3A and B, this was indeed the case; when used together, MP1 and EJs displayed the best antibiofilm effect against LMGT 7660, leading to a ∼98 % decrease in biofilm-associated metabolic activity (*P*_Ctrl:MP1/EJs_ = 1.8e-10), against the 83 % of MP1 (*P*_MP1/EJs:MP1_ = 0.016, two-sample *t*-test) and the ∼60 % of EJs 40 μg (*P*_MP1/EJs:EJs40_ = 0.0003, two-sample *t*-test).

**Fig. 4:**
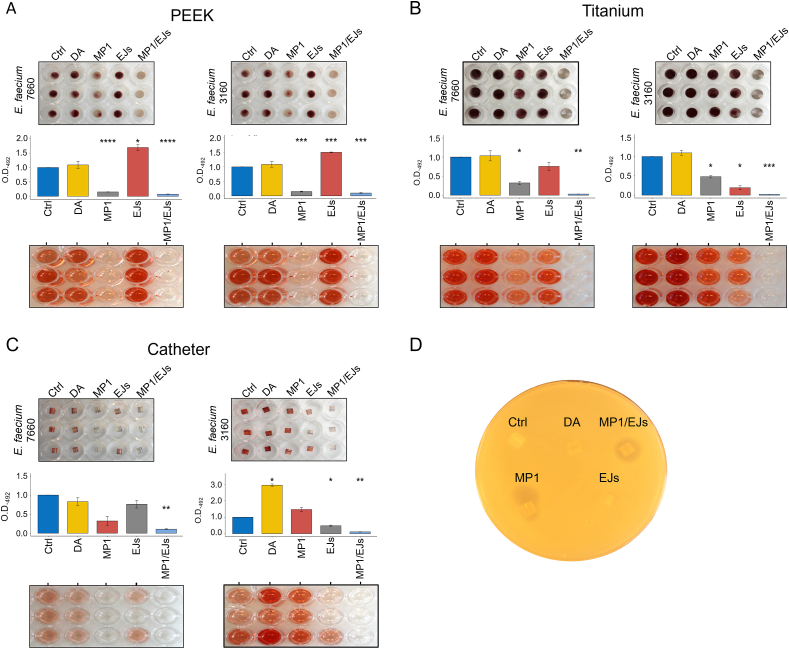
The combination MP1/EJs inhibits biofilm formation by LMGT 7660 and 3160 on different materials. (A) Representative images of biofilms produced by *E. faecium* 7660 and 3160 on PEEK discs left uncoated (Ctrl) or coated with the indicated proteins. The biofilm-associated metabolic activity was quantified as described in Fig. 3B. (B and C) Same as in panel 'A' but the materials were titanium discs (B) or silicone catheter segments (C). Shown are the average values (±s.d.) of at least three independent experiments. (D) Spot-on-lawn assay showing the diffusion of bacteriocins from silicone (Foley) catheters fragments deposited on a bacterial lawn of *E. faecium* LMGT 7660. *p < 0.05; **p < 0.01; ***p < 0.001; ****p < 0.0001.

The results obtained with the stainless steel led us to explore whether the bacteriocin coating could have a similar antibiofilm function on different materials of medical interest. For these assays, both *E. faecium* LMGT 3160 and LMGT 7660 were used (Fig. S2). As can be seen in Fig. 4A–C and in line with the results obtained with stainless steel, MP1 and EJs displayed an antibiofilm activity which was indeed further improved when the two bacteriocins were used in combination, reaching inhibition percentages close to 90 % for silicone (Foley) catheters (89 % for both strains) and PEEK (92 % LMGT 7660; 90 % LMGT 3160) or close to 100 % in the case of titanium discs (97 % LMGT 7660; 98 % LMGT 3160). It is also interesting to note that, while the activity of the MP1/EJs combination was consistent on all materials tested, that of the single bacteriocins displayed a higher variability (Fig. 4A–C). Furthermore, at least in the case of coated silicone catheters, the bacteriocins exerted their antimicrobial activity in the surrounding environment likely due to a slight release of MP1 from the catheters (Fig. 4D and Fig. S5).

**Fig. 5:**
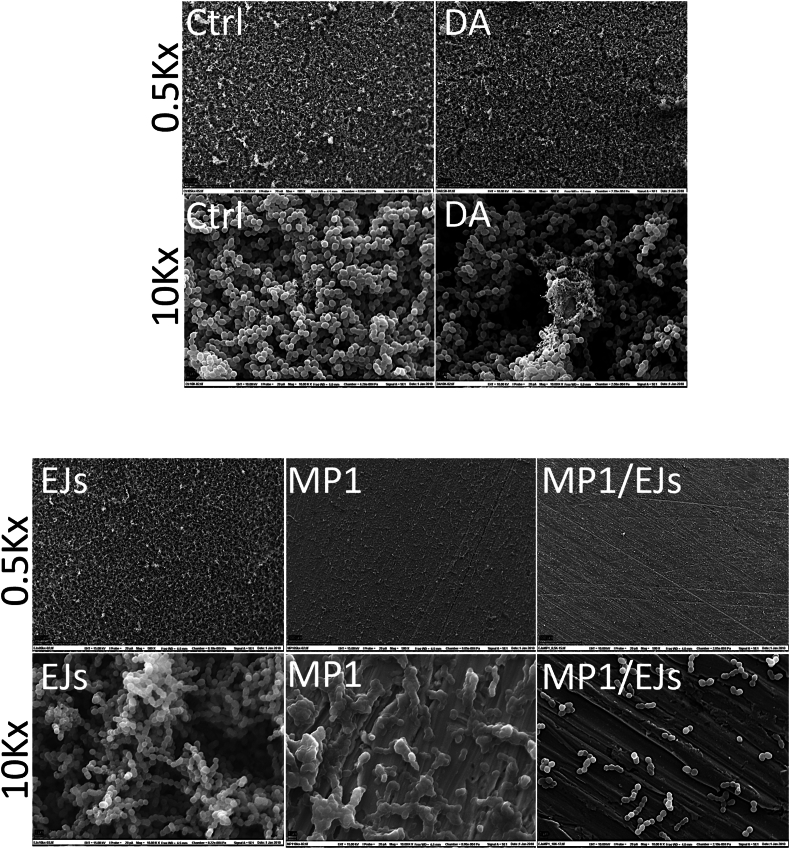
Visualization of the antibiofilm activity of the MP1/EJs combination by scanning electron microscopy. Titanium discs left uncoated (Ctrl) or coated with the indicated proteins were incubated with *E. faecium* LMGT 7660. Images were taken at a magnification of 500× (0.5Kx) or 10,000x (10Kx). The white bars in the lower left corner of each panel indicate 100 μm in the 0.5Kx images and 1 μm in the 10Kx images.

The combination of MP1 and EJs appeared to be most efficient on the titanium disks, where the single bacteriocin treatments were clearly inferior to the combination (Fig. 4B). To further characterize this, we analyzed the biofilms formed by LMGT 7660 on treated titanium discs by scanning electron microscopy (Fig. 5). As can be seen, control and polydopamine-coated discs allowed the formation of thick biofilms on their surface. While EJs seemingly failed to inhibit biofilm formation, the biofilm was greatly altered on the MP1-coated discs, with the bacterial cells appearing morphologically altered. In agreement with the metabolic activity data, the combination EJs/MP1 coating displayed clear inhibitory effects on cell attachment and biofilm formation, although a few cells appeared to adhere to the material's surface.

We also noted that when the coated catheters were placed on bacterial lawns on agar plates, the bacteriocins exerted their antimicrobial activity in the surrounding environment, suggesting a slight release of MP1 from the material (Fig. 4D). Analytical HPLC was then used to determine the release of MP1 to the buffer after incubation of MP1-coated catheter in phosphate buffer over a period of 26 h (Fig. S5). This assay indicated that only tiny amounts of MP1 was released from the catheter and that the release was almost completed over the initial few hours.

Therefore, these data show that bacteriocins can be effective anti-fouling agents against antibiotic resistant strains when used to coat different materials of medical interest.

### 2.3 The bacteriocins EJs and MP1 released from materials are not cytotoxic to eukaryotic cells

Indwelling medical devices are necessarily in close contact with anatomical structures and living tissues. We were therefore interested to address whether the EJs and MP1 could have any cytotoxic effect on eukaryotic cells. The cytotoxicity of the bacteriocins themselves was first tested in MG63 and Ovcar-8, human osteosarcoma and ovarian cancer cell lines, respectively. The results of these assays are shown in Fig. S6 and, as can be seen, while none of the bacteriocins had cytotoxic effects on MG63 cells up to 80 μg ml^−1^ (log_10_ = 2, Fig. S6), Ovcar-8 cells displayed an increased sensitivity to MP1 with an IC_50_ = 44.1 μg ml^−1^, which, nevertheless, is slightly higher than the concentration of bacteriocin used in the coating reactions (40 μg ml^−1^).

**Fig. 6:**
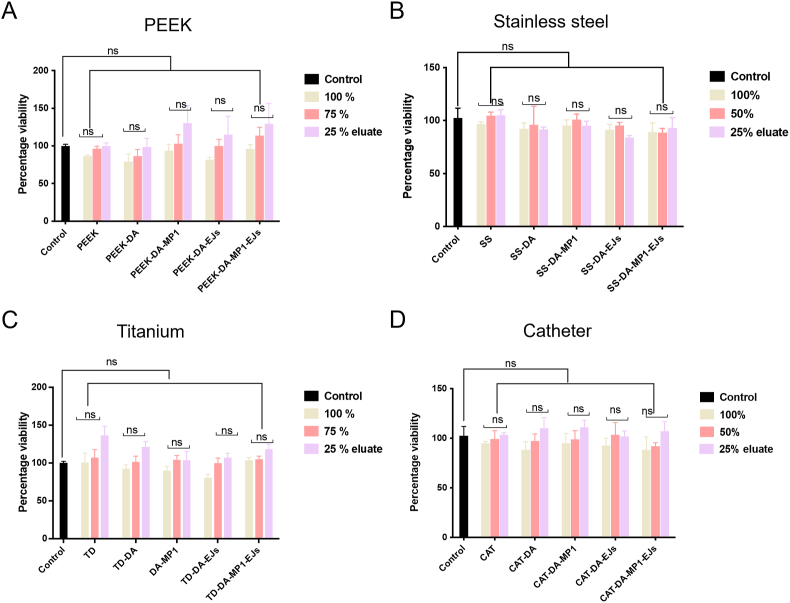
MP1 and EJs are not cytotoxic for MG63 human osteosarcoma cells. (A) test comparison of PEEK elution samples, where 100 %, 50 %, and 25 % of the initial eluate were used. (B, C and D) same as in panel 'A′ but the peptides were eluted from stainless steel, titanium discs and silicone (Foley) catheter segments.

Second, we tested if the bacteriocins eluted from implant materials would show cytotoxicity. The eluates from the four implant materials were added to the culture medium of osteosarcoma MG63 cell line and their effect on cell viability was measured. It was observed in the comparison plot that there was no statistically significant difference between the negative control and bacteriocins alone or in combination for PEEK (Fig. 6A), stainless steel (Fig. 6B), titanium discs (Fig. 6C) or silicone catheter (Fig. 6D). Hence the four peptides-modified implant material samples were confirmed to be non-cytotoxic.

Taken together, these data indicate that the bacteriocins used in this study are weakly or non-cytotoxic to eukaryotic cells.

## 3 Discussion

The ability of bacteria to adhere to biotic and abiotic surfaces and form biofilms has evolved as passive defense mechanism against environmental challenges. Advances in the understanding of biofilms in bacterial pathogenicity and the increased use of exogenous materials in various applicative aspects of medicine, has led to extensive research in strategies to prevent biofilm formation on a variety of materials. Among these, AMPs have been widely explored as potential preventive antibiofilm agents in various fields. Antimicrobial peptides, such as bacteriocins, retain antimicrobial activity at low concentrations against antibiotic-resistant bacterial strains and, as in the case of nisin, they have been recognized generally safe for human use. Nisin is perhaps the best studied bacteriocin and, because of its safety, it has been widely studied in a variety of different applications [[42], [43], [44]], including as antibiofilm coating on stainless steel and other materials [[45], [46], [47], [48], [49], [50]]. In our hands nisin Z, a natural isoform of nisin A with overlapping structure and activity [51,52], failed to significantly reduce the biofilm formation on stainless steel by *E. faecium*. This is in contrast with previous studies where nisin A was used as antibiofilm agent to functionalize stainless steel coupons [50,53]. These studies, however, assessed the biofilm inhibitory effects of nisin against *Listeria ivanovii* and *Bacillus subtilis* and not *E. faecium*. In addition, different technologies to bind nisin to the stainless steel surface were used.

Based on the initial analysis, we here investigated the activities of four implant materials coated with the thiopeptide bacteriocin (MP1) and a synthetic non-translationally modified enterocin (EJs) via a polydopamine mediated binding. Previous studies have shown that enterocins and thiopeptides can affect the viability of biofilm-associated bacterial cells [9,13,36,[54], [55], [56], [57]]. However, only a single study has assessed enterocins for their ability to prevent the formation of biofilms on glass and stainless steel [58]. Al Atya and colleagues [58] showed that enterocins DD28 and DD93, produced by *E. faecalis* strains, were able to delay the formation of MRSA biofilms alone or in combination with erythromycin. It is interesting to note that similarly to these results, our experiments also showed that EJs and MP1 alone were only able to slightly reduce the biofilm associated metabolic activity, possibly suggesting a similar delaying effect in biofilm formation. Enterocin EJs, which was designed in our lab, is a truncated version of enterocin EJ97 (lacking the first seven amino acids) [36]. Enterocin EJ97 belongs to the LsbB bacteriocin family along with LsbB, EntK1 and EntQ [59]. Similar to other enterocins, the LsbB-related group exerts its antimicrobial activity on target cells by binding to a membrane-protein receptor eventually resulting in cell death [14,57,60]. Functional studies showed that, at least against *Staphylococcus pseudintermidius*, EJs retains an activity similar to its full-length counterpart [36].

Unlike enterocins, the antimicrobial mechanism of action of MP1 and other thiopeptides relies on the inhibition of bacterial protein synthesis by direct interaction with bacterial ribosomal proteins [61]. Therefore, for the thiopeptide bacteriocin to bind the target ribosomal protein it must diffuse within the cytoplasm of bacterial cells. Our data indeed suggest that MP1 is slightly released from the materials' surface, at least in the case of coated silicone (Foley) catheters (Fig. 4D and Fig. S5) and is thus free to enter the *E. faecium* cells to exert its antimicrobial activity. Similarly, peptides on the dopamine-coated titanium were previously found to be largely stable although a portion of them could be released in the longer incubation period [62]. MP1 and EJs appear to have the best anti-biofilm properties against *E. faecium* when used in combination. This is also evidenced by our scanning electron microscopy analysis, where only a few cells were able to adhere to the double coated titanium surface. Based on our experiments, we have not been able to conclude whether these cells are still viable and/or whether they represent persister cells. Furthermore, the mechanism of antimicrobial interaction between MP1 and EJs has yet to be fully elucidated. he two bacteriocins have different antimicrobial mechanisms of action may confer a more robust antimicrobial activity by increasing the bacterial cell fitness cost required to accumulate critical mutations, prevent mechanisms of resistance. Indeed, a strong synergy between these two bacteriocins were shown when tested with checkerboard assays (Table S2). It is also important to mention that the antimicrobial activity of the bacteriocins might be separated from their antibiofilm function, since the latter could also be linked to an inhibition of bacterial adhesion on the functionalized material by the bacteriocins. Thus, whether the anti-biofilm activity observed here are caused by reduced adhesion, cell killing or a combination of these, remains to be determined – and also whether these effect vary between the different materials.

To further develop the coating implants with these bacteriocins for clinical applications further, more knowledge about bioavailability and stability of these peptides in relevant conditions would be needed, since liquid flow and presence of protease limit the effect of the coated materials. Moreover, *in vivo* studies using the animal models need to be carried out. Interestingly, several AMPs bound to metallic implant materials such as titanium and stainless steel, showed promise and efficacy in animal models in control of biofilm formation on the implant surfaces [19].

In conclusion, this study demonstrates that bacteriocins can be used to functionalize materials of medical interest to prevent biofilm formation by antibiotic-resistant bacterial pathogens. Further work will be aimed at expanding the plethora of pathogenic bacteria to be tested on bacteriocin-coated materials and to define the antibiofilm activity of bacteriocin coatings in near-physiologic conditions (e.g., using urine or blood as medium). Furthermore, this technology will have to be rigorously tested in preclinical animal models before it can approach clinical applications. However, given the general lack of studies investigating strategies to prevent *E. faecium* biofilm formation on indwelling medical devices, we hope that our study can contribute to the management of hospital-acquired infection brought about by this emerging nosocomial pathogen.

## 4 Materials and methods

### 4.1 Implant materials and bacteriocins

PEEK discs with a diameter of 0.7 cm were purchased from PCBway (China), titanium discs with diameters of 1 cm were manufactured by Baoji Minghai Titanium Industry Co.,Ltd (China) and stainless steel discs were purchased from Wuhan Steel factory (China). 100 % polysilicone catheters, with a tube diameter of 0.45 cm, were purchased from ConvaTec Limited, UK. The catheter tube was cut into small cylindrical tubular segments of 0.5 cm in length. The stainless-steel discs were polished using grade 600# sandpaper so as to obtain maximum adherence of peptides. The bacteriocins EntK1, EntEJ97, EJs used in the study were custom synthesized by Pepmic Co., Ltd (China) with purity ≥95 %. Micrococcin P1 and nisin Z were produced as described previously [13,63]. Dopamine hydrochloride and Tris-HCl were obtained from Sigma Aldrich, St Louis, USA.

### 4.2 Dopamine treatment

All the materials were placed in 24-well cell plates and washed three times with MilliQ water (Millipore) in an ultrasound water bath (Fisher Scientific, UK) for 7 min each wash. Subsequently, PEEK and silicone catheter segments were treated with a 1 mg ml^−1^ dopamine solution in 10 mM Tris-HCL buffer (pH 8.5, Sigma-Aldrich, St. Louis, USA) at 37 °C, whereas a 0.04 mg ml^−1^ dopamine solution was used for metals (stainless steel and titanium discs). The dopamine was left in contact with the materials for 24 h with gentle shaking (150 rpm on a rotator shaker) at room temperature (RT) protected from light. Following the reaction, the excess polymeric dopamine was eliminated by rinsing the materials three times with MilliQ water and then washing them in an ultrasound water bath (Fisher Scientific, UK) for 20 s. The materials were further washed by putting them on a rotator shaker for 10 min and at 150 rpm for three times and then air-dried. All the materials were then placed in sterile 24-well plates and stored at RT until further treatment with bacteriocins.

### 4.3 Surface coating with antimicrobial peptides

Solution of MP1 (20 mg ml^−1^ in DMSO), and EJs (20 mg ml^−1^ in water) were prepared. Initially, 20 μl of 10 mM Tris HCl buffer, pH 8.5 were added exactly onto the top of the PEEK, stainless steel and titanium materials to be treated with the peptides. Implant material samples with a small amount of Tris buffer sited on the top surface were then treated with 2 μl (20 mg ml^−1^) of each peptide separately to increase the initial concentration of peptides for coating. For combination MP1/EJs coatings, a mixture of both the peptides (2 μl each) was added to the sample surface along with the Tris HCl and mixed lightly. The materials were left on the rotator shaker at 150 rpm for 3.5 h. Subsequently, 1 ml of 10 mM Tris HCl, pH 8.5 was added to the well plates to dilute the peptide solutions to 40 μg ml^−1^ for coating overnight. For silicone catheter samples, they were directly immersed in 1 ml 10 mM Tris-HCl buffer, pH 8.5, and 2 μl (40 mg ml^−1^) of MP1 and EJs were added separately or in combination to reach a final concentration of 40 μg ml^−1^ of MP1 and/or EJs. For surface coating of stainless steel with Nisin Z, EJ97 and EntK1, DA-coated stainless steel discs were directly incubated with 40 μg ml^−1^ of those peptides in 1 ml Tris-HCl buffer as above. Coating reactions were left to proceed on a rotator shaker at 150 rpm for 20 h protected from light. The materials were then washed with 2 ml distilled water in an ultrasound water bath for 20 s (three times) and washed again by placing in 2 ml distilled water on a rotator shaker at 150 rpm for 10 min (three times). All the materials were then air-dried and stored at RT until further use.

### 4.4 Stability of coatings of MP1 on silicon catheter

MP1-coated silicon catheter was incubated with 0.5 ml of 50 mM phosphate buffer, pH7.4 over 26 h, at the time point of 2, 6 and 26 h, 50 μL samples (3 times) were taken and injected into high performance liquid chromatography system with an analytical column coupled with a UV–vis detector (Phenomenex, UK; 5 μm particle size, 4.6 × 250 mm). For the control of MP1, 10 μL of MP1 (40 μg ml^−1^ in phosphate buffer with 0.2 % DMSO) was injected. The mobile phase A was distilled water, and B was 100 % methanol. The mobile phase started with 40 % B and rose to 100 % B over 10 min and was maintained at 100 % B for 5 min at a flow rate of 1 mL/min at 350 nm absorbance.

### 4.5 Contact angle measurements

A Milli-Q (Millipore) water drop of 1 μl was released on the surface of the modified materials and a water contact angle analyzer (Biolin Scientific) equipped with the One Attention software was used to record contact angles within 20 s. The mean of five measurements was reported.

### 4.6 X-ray photoelectron spectroscopy (XPS)

The XPS analysis was performed by the Warwick Photoemission Research Technology Platfortm, University of Warwick (UK). The samples were attached to an electrically conductive carbon tape and loaded onto the sample bar. This was then loaded into a Kratos Axis Ultra DLD spectrometer with a base pressure below 1 × 10^−10^ mbar. In the primary analysis chamber, XPS measurements were carried out while the samples were illuminated by a monochromated Al K X-ray source (hv = 1486.7 eV). The measurement was taken at a take-off angle of 90° at RT. The core level spectra were recorded using a pass energy of 20 eV (resolution approx. 0.4 eV), from an analysis area of 300 mm × 700 mm. Prior to the start of the studies, the spectrometer work function and binding energy scale were calibrated using the Fermi edge and 3d5/2 peak recorded from a polycrystalline Ag sample. In order to compensate for the accumulation of positive charge on the surface during the photoemission process on the catheter and PEEK samples, a charge neutralizer was used to flood the surface with low energy electrons. As a result, the binding energy scale has to be referenced to the peak of C–C/C–H in the C 1s area at 284.8 eV. The data were analyzed with the CasaXPS software utilising mixed Gaussian-Lorentzian (Voigt) line shapes and Shirley backdrops. In order to calculate the detection efficiency throughout the whole binding energy range for compositional analysis, clean metallic foils were used to estimate the analyser transmission function. Microsoft Excel was then used to plot the readings that were acquired.

### 4.7 Bacterial strains and culture conditions

The *E. faecium* strains used in this study are clinical isolates kindly gifted by Prof. Rob J. L. Willems (University Medical Centre Utrecht, Utrecht, The Netherlands) [64]. Unless otherwise stated, *E. faecium* strains were cultured in brain heart infusion (BHI) medium (Oxoid) at 37 °C under aerobic conditions.

### 4.8 Verification of the biofilm formation abilities

*E. faecium* strains were inoculated in 5 ml of sterile brain heart infusion (BHI) and cultured as described above. The bacterial cultures were then adjusted to O.D._600_ = 0.5 and diluted 1/10 in 90 μl of tryptic soy broth (TSB - Millipore) supplemented with 1 % d-glucose monohydrate (Millipore) in 96-well plates and incubated for an additional 48 h in static conditions at 37 °C. To minimize the variation between the different independent experimental replicas, each strain was inoculated in all the wells of a full plate column. The biofilm formation abilities of the different strains were then assessed using the crystal violet method as described previously [9].

### 4.9 Biofilm assays on coated materials

Biofilms of the *E. faecium* strains LMGT 7660 and LMGT 3160 were allowed to form as described above on the surface of unmodified (Ctrl) or DA/DA-bacteriocin-modified materials in 24-well plates. Residual planktonic cells were eliminated by submerging the discs or catheter segments in a 0.9 % NaCl solution and the materials were then incubated in the nutrient broth a 0.025 % solution of 2,3,5-triphenyltetrazolium chloride (TTC – Sigma-Aldrich) for an additional 16h. The materials were then placed in fresh 24-well plates, photographed, and then incubated overnight (O/N) in a 70:30 ethanol: acetone mixture to extract the red formazan accumulated within the metabolically active cells. Optical density readings at 592 nm (OD_592_) were then used to quantify the residual biofilm-associated metabolic activity.

### 4.10 Synergy assessment

Assessment of synergy between MP1 and EJs was done using checkerboard assays as described previously [13,65]. The fractional inhibition concentration (FIC) was calculated as described [13], and the effects were considered synergistic when FIC ≤0.5 [66].

### 4.11 Scanning electron microscopy

*E. faecium* LMGT 7660 biofilms were grown on coated titanium discs as described above. Subsequently, biofilms were carefully washed twice in phosphate-buffered saline and then fixed in 3 % glutaraldehyde O/N. The biofilms were dehydrated in increasing alcohol series of 30, 50, 70, 90, 96 % ethanol for 10 min each, followed by 4 × 10 min in 100 % ethanol. The samples were then subjected to critical point drying and sputter-coated with a palladium–gold thin film before examination by a scanning electron microscopy (SEM) system (Zeiss) at 15 kV.

### 4.12 Eukaryotic cell culture conditions and cytotoxicity tests

Human osteosarcoma cells (MG63) and Human ovarian cancer cells (Ovcar-8) were maintained in Roswell Park Memorial Institute (RPMI 1640, Lonza) medium supplemented with 10 % FBS and 1 % PenStrep antibiotic mix (Sigma Aldrich) and in Dulbecco's Modified Eagle Medium (DMEM) supplemented with 10 % FBS and 1 % PenStrep antibiotic mix, respectively, at 37 °C with 5 % CO_2_. Cells were routinely passaged when they were ∼80 % confluent.

The 3-(4,5-dimethylthiazol-2-yl)-2,5-diphenyltetrazolium bromide (MTT) cytotoxicity assay was carried out in accordance with the ISO 10993-5 2009 standard (Biological evaluation of medical devices – tests for *in vitro* cytotoxicity) to evaluate the toxicity of the eluates of the implant materials. Control implant materials, DA-coated and peptide-modified materials after UV irradiation for 30 min were submerged in DMEM supplemented with 10 % FBS (with a ratio of 1 ml per 3 cm^2^ of implant surface, 0.5 and 1.0 ml for PEEK and titanium disc, respectively) in a 24-well plate and incubated for 24 h at 37 °C to elute the respective coating moieties. MG63 cells and Ovcar-8 were trypsinzated and 1 × 10^4^ cells were transferred to the appropriate wells of a 96-well microplate in a total volume of 100 μl/per well of their respective culturing media. Cells were then left to grow for 24 h at 37 °C and 5 % CO_2_. The culture media were then replaced by the eluates at concentrations of 100, 50 or 25 % (vol/vol) diluted in culture medium, plain medium (negative control), prior to incubating the cells for an additional 24 h. To quantify cell viability, the culture media were removed and 50 μl of MTT reagent (Thermo Fisher Scientific Inc., Waltham, MA, USA) with a concentration of 0.5 mg ml^−1^ in DMEM was added to each well and incubated for 4 h at 37 °C. After decanting MTT solution, the blue formazan product was solubilized with 100 μl of dimethylsulfoxide (DMSO, Fisher Scientific, UK), and was finally centrifuged at 150 rpm for 15 min (CN-15, Hsiang Tai Machinery Industry Co., Ltd., New Taipei City, Taiwan). With the aid of a spectroscopic plate reader, the absorbance at 570 nm is determined (multi-mode microplate reader BioTEK Synergy 2, USA). The difference between the test item's viability and the blank was calculated using the formula below:

Viability% = 100 * (OD570e/OD570b),

where OD570e denotes the average optical density of 100, 50, and 25 % of the test item extracts, and OD570b denotes the average optical density of the blanks.

The further determination of IC_50_ of the bacteriocins alone used in MG-63 and Ovcar-8 cells were performed according to the published procedure [67,68].

### 4.13 Statistical analysis

All graphical representations and statistical significance analyses involving the data collected from the biofilm assays were performed using the R-studio software version 2023.06.0 + 421. The statistical significance for the contact angle measurements and the cytotoxicity assays were calculated and tabulated using GraphPad Prism version 9 (GraphPad Software, Inc., La Jolla, USA). Statistical significance was set at a p-value <0.05. Shapiro-Wilk and Levene's tests were done to analyze the normality of the distribution and the homogeneity of variance.

## Conflict of interests

The authors declare no conflict of interests.

## CRediT authorship contribution statement

**Christian Kranjec:** Writing – review & editing, Writing – original draft, Visualization, Methodology, Investigation, Formal analysis, Data curation, Conceptualization. **Jills Puthiaparambil Mathew:** Methodology, Investigation. **Kirill Ovchinnikov:** Resources. **Idowu Fadayomi:** Investigation. **Ying Yang:** Writing – review & editing, Funding acquisition. **Morten Kjos:** Writing – review & editing, Conceptualization. **Wen-Wu Li:** Writing – review & editing, Visualization, Supervision, Project administration, Funding acquisition, Conceptualization.

## Declaration of competing interest

The authors declare the following financial interests/personal relationships which may be considered as potential competing interests:

Wen-Wu Li reports financial support was provided by Research England. Kirill Ovchinnikov has patent Synergistic bacteriocin compositions pending to Assignee. Christian Kranjec has patent Synergistic bacteriocin compositions pending to Assignee. If there are other authors, they declare that they have no known competing financial interests or personal relationships that could have appeared to influence the work reported in this paper.

## Data Availability

Data will be made available on request.

## Abbreviations

CA: contact angle
XPS: x-ray photoelectron spectroscopy
EJs: enterocin EJ97-short
MP1: micrococcin P1
EntEJ97: enterocin EJ97
EntK1: enterocin K1
EntQ: enterocin Q
PEEK: polyetheretherketone
TD: titanium discs
SS: stainless steel
DA: (poly)dopamine
VRE: vancomycin-resistant *Enterococcus*
MDR: multidrug-resistant
AMPs: antimicrobial peptides
