# Supplementary material for: A bacteriocin-based coating strategy to prevent vancomycin-resistant Enterococcus faecium biofilm formation on materials of interest for indwelling medical devices
Source: Biofilm. 2024 Jul 3;8:100211. doi: 10.1016/j.bioflm.2024.100211 (PMC11282937; doi:10.1016/j.bioflm.2024.100211)
Supplement: Multimedia component 1 [file mmc1.pdf]

Supplementary figures

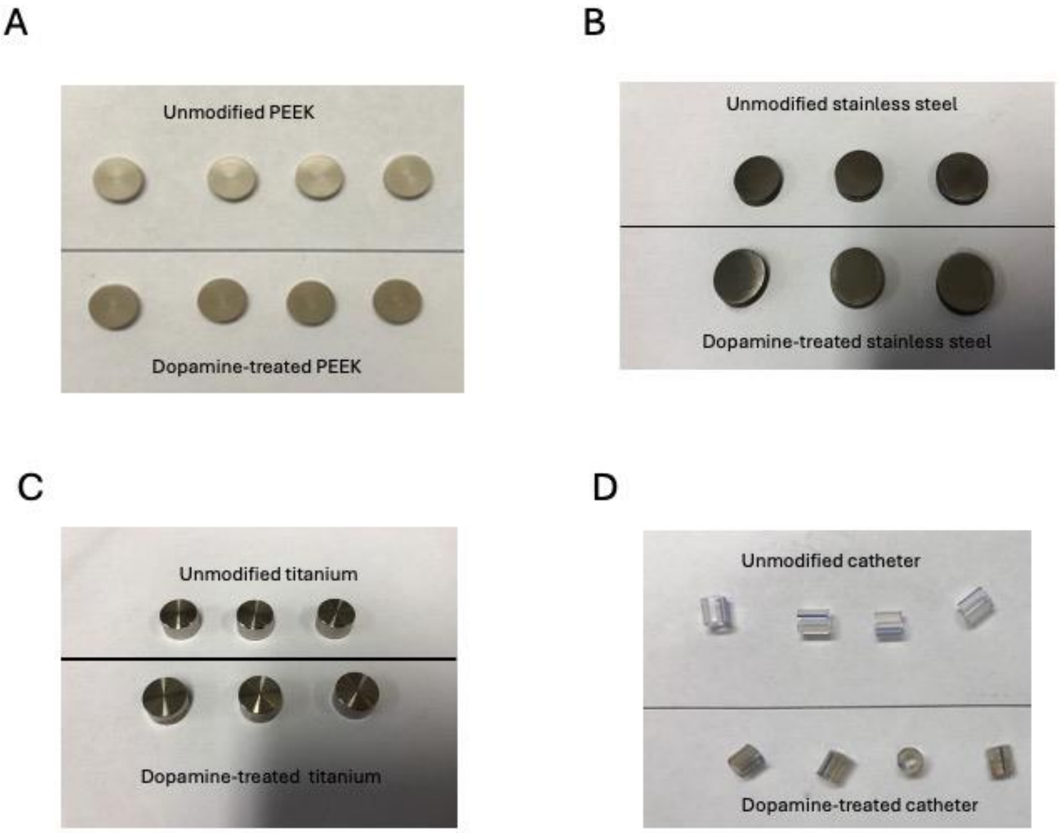

Figure S1. The coating with polydopamine modifies the color of the treated materials. Representative pictures of PEEK discs (A), stainless steel discs (B), titanium discs (C) and silicone catheter segments (D) before and after the coating reaction with dopamine.

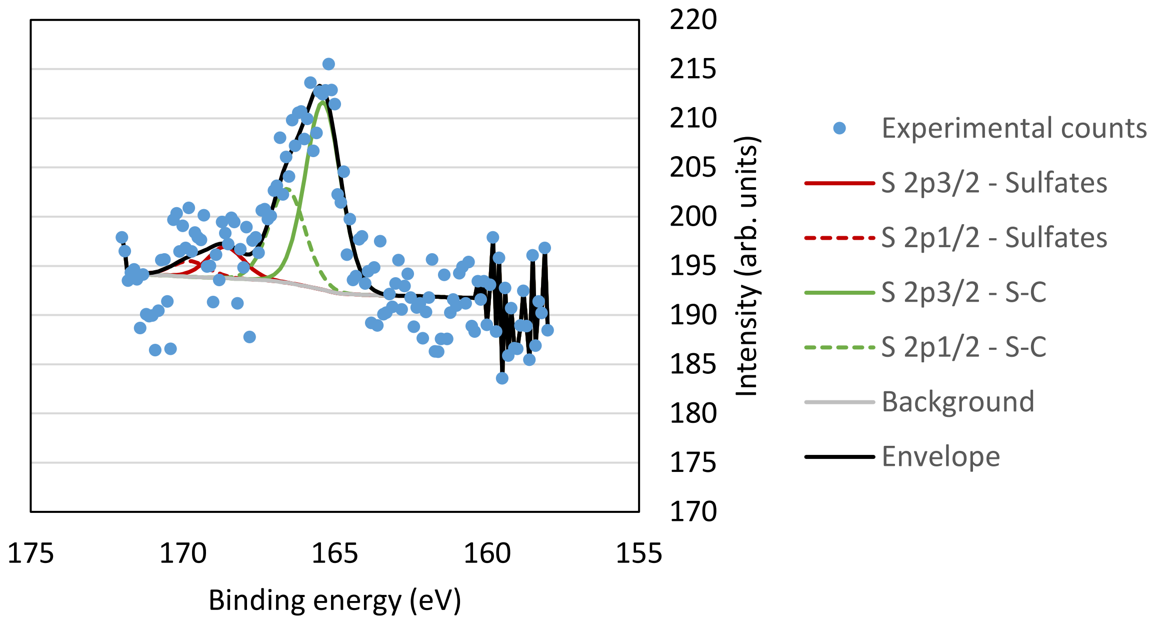

Figure S2. XPS spectra of the S2p core level region of MP1-DA-Titanium sample (showing the presence of very likley sulfur element (S2p) on the titanium surface through peak fitting. The experimental counts (blue dots) approximate well with the envelope line (solid black line) which is the sum of the four likley component peaks (S 2p 3/2 - S-C, S 2p1/2 S-C, S 2p 3/2 - sulfates, S 2p1/2 sulfate) followed by the addition of the Shirley background line. See Major et al. (2020) *J. Vac. Sci. Technol. A* 38, 061203 for details on the procedure

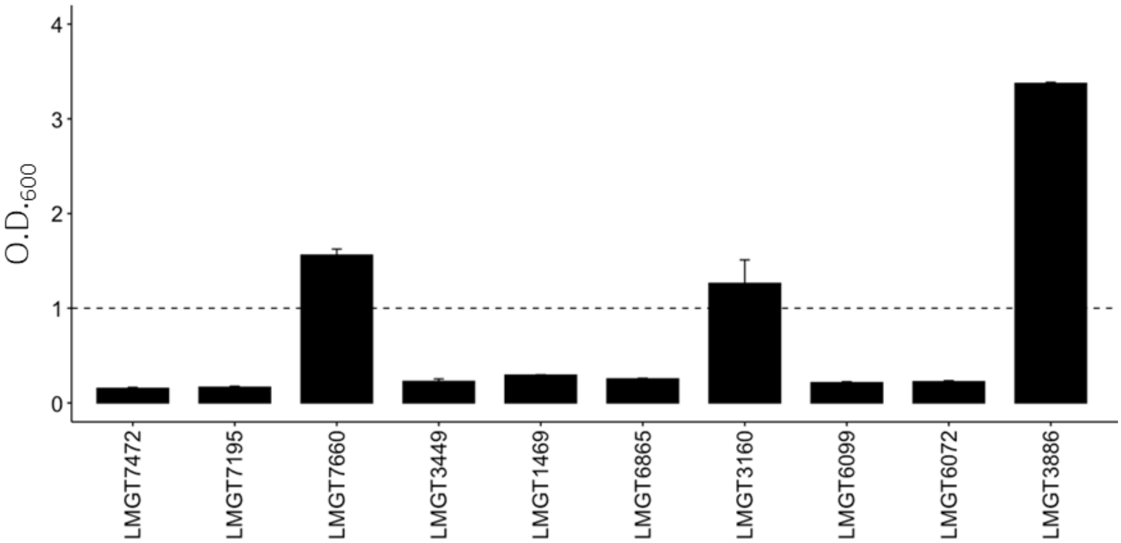

Figure S3. Biofilm formation ability of nine *E. faecium* strains from our collection. Biofilms for the indicated strains were allowed to grow for 48h prior of being subjected to crystal violet staining. The amount of dye bound to the cells is an indirect measure of the biofilm-forming abilities and was quantified by optical density readings at 600 nm ($O.D._{600}$) for each strain. The bar chart shows the average values (± s.d.) obtained from three independent experiments. *Staphylococcus aureus* LMGT 3886 was used as positive control for biofilm formation. The broken line shows the cut-off O.D. value (O.D. = 1) under which the strains were considered non biofilm formers.

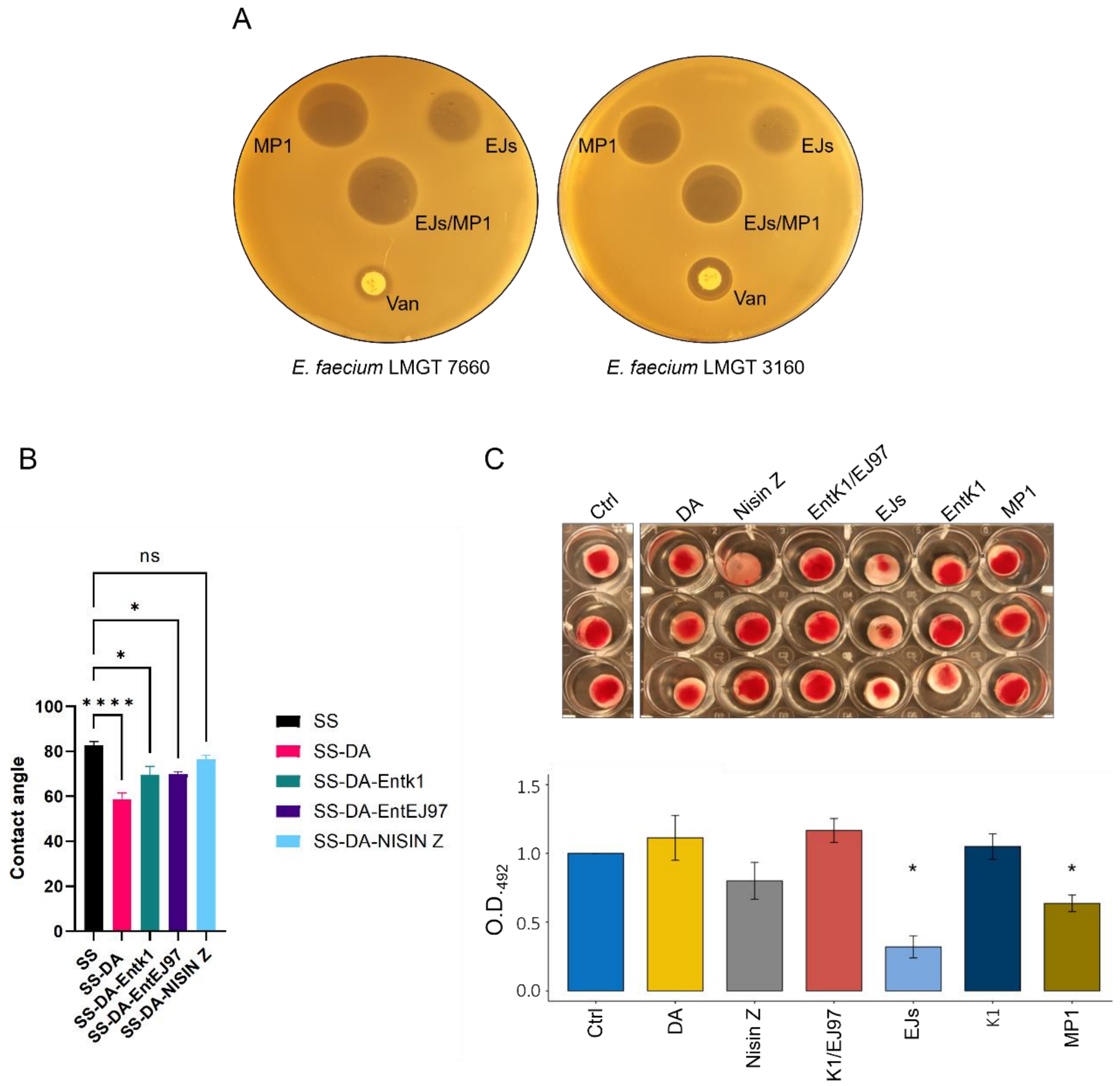

Figure S4. Antibiofilm and antimicrobial effects of bacteriocins against *E. faecium*. (A) Spot-on-lawn assay showing the effects of MP1, EJs or a combination of the two in comparison to vancomycin (Van) on the indicated *E. faecium* strains. EJs = 10 μg; MP1 = 10 μg; Van = 5 μg. (B) Water contact angle measurements performed on stainless steel discs left uncoated (SS) or coated with polydopamine (SS-DA) or DA and the indicated bacteriocins. Statistical significance was analyzed by pairwise comparison of each group with the unmodified SS control. (C) Representative image *E. faecium* 7660 biofilm-associated metabolic activity on SS discs left uncoated (Ctrl) or coated with the indicated proteins. The bar-plot shows the colorimetric quantification of the metabolic activity after the red formazan elution. The metabolic activity is expressed as $O.D._{492}$ fold change relative to the uncoated SS discs (Ctrl). Shown are the average values (± s.d.) of at least three independent experiments. Statistically significant differences in the average metabolic activity across the groups were analyzed with the One-way ANOVA test. Post-hoc pairwise comparisons relative to Ctrl were performed using the two-sample t-test. The standard deviation is also shown. $^*p < 0.05$.

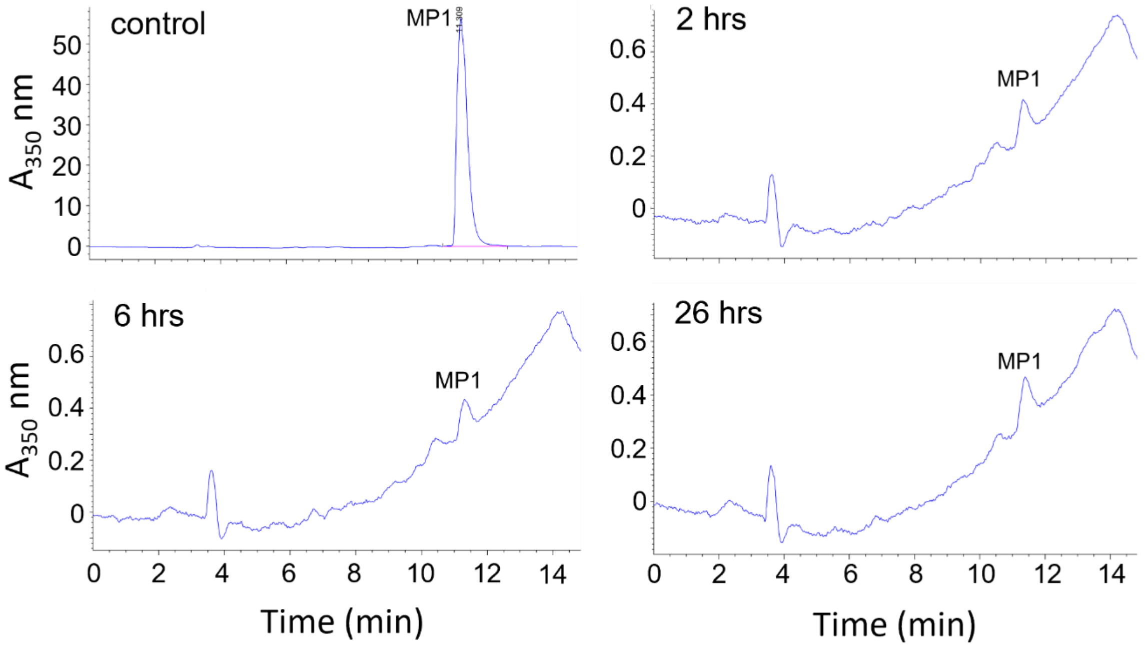

Figure S5. HPLC chromatograms of control MP1 (A), and its release of MP1 from MP1-DA-Catheter sample after incubation in phosphate buffer for 2 h (B), 6 h (C) and 26 h (D) detected at absorbance of 350 nm. The peak intensity of MP1 for 2h, 6h and 26 (0.4) is very low compared to that (50) of the control (by injection of 10 µL of 40 µg/ml MP1, equal to 400 ng of MP1) does not change, indicating minimal or no release of MP1 after 2h of incubation.

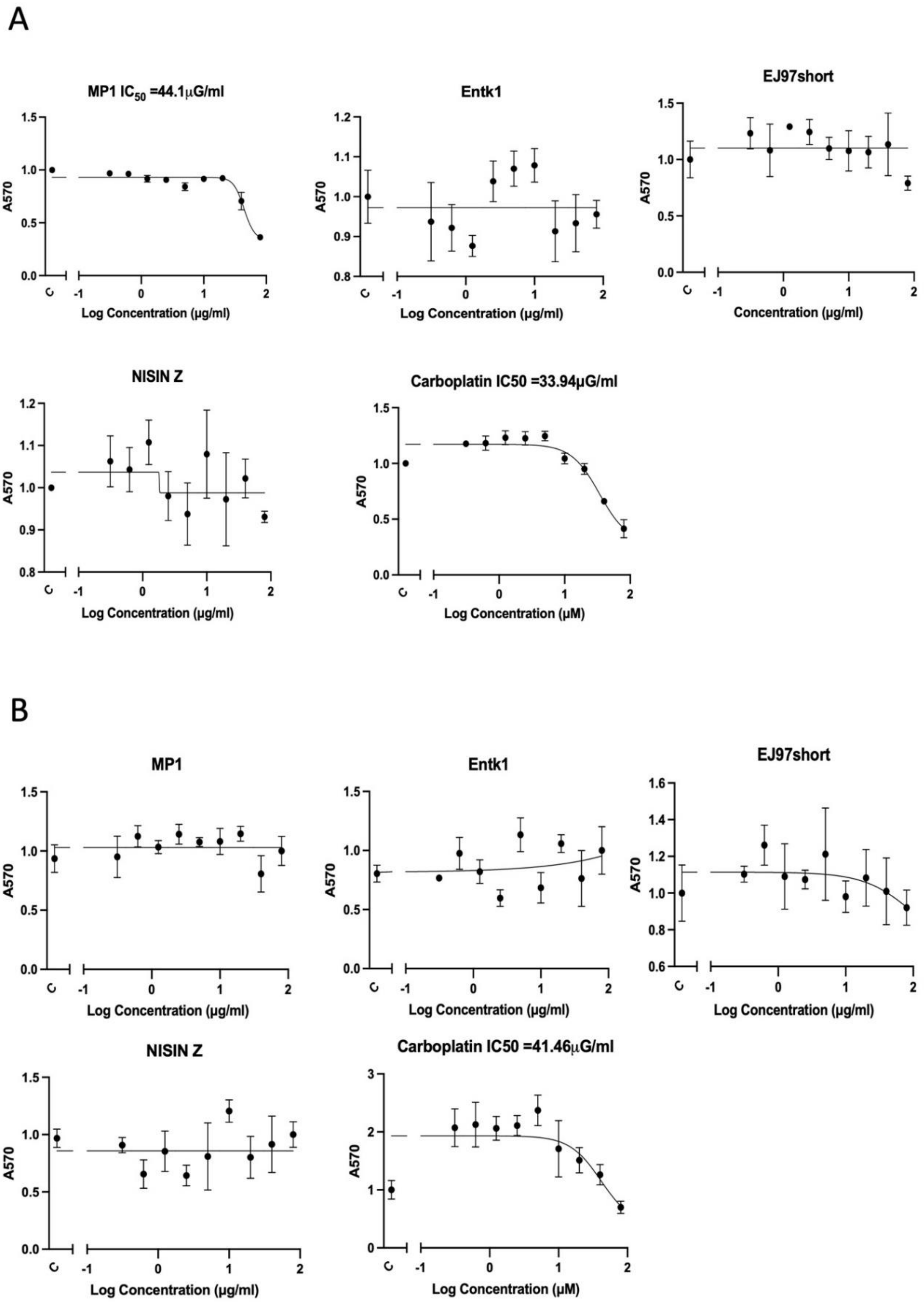

Figure S6. The bacteriocins tested are not cytotoxic for eukaryotic cells. (A) cytotoxicity test on MG63 cells of the indicated bacteriocins (MP1, EntK1, EJs, and Nizin Z) and carboplatin. (B) Same as in panel `A` but the cytotoxicity test was performed using Ovcar-8 cells.

Supplementary tables

Table S1. Summary of presence/absence of peaks N1s and S2p in the XPS spectra (related to main Figure1).

Coating	Antimicrobial	Peaks[a]	
		**N1s**	**S2p**
PEEK	Control	-	-
	DA	+	-
	MP1	+	+
	EJs	+	-
	MP1-EJs	+	+
Stainless steel	Control	-	-
	DA	+	-
	MP1	+	+
	EJs	+	-
	MP1-EJs	+	+
Titanium	Control	-	-
	DA	(+)	-
	MP1	+	(+)
	EJs	+	-
	MP1-EJs	+	(+)
Silicone catheter	Control	-	-
	DA	-	-
	MP1	-	(+)
	EJs	-	-
	MP1-EJs	-	(+)

[a] Signs denote absence (-) or presence of strong or weak peaks (+ and (+), respectively) in the XPS spectra (Figure 1).

Table S2. Synergy assessment between MP1 and EJs against two strains of *E. faecium*.

MIC[a] (μg/ml)	Strains *E. faecium* LMGT 7660	Strains *E. faecium* LMGT 3160
MP1	> 25	> 25
EJs	> 25	> 25
MP1 / EJs (in combination)	0.1 / 1.6	0.2 / 0.8
FIC[b]	< 0.07	< 0.04

[a] Minimum inhibitory concentration.
[b] Antimicrobial combination considered synergistic if FIC < 0.5.
